# Cancer Cachexia Induces Preferential Skeletal Muscle Myosin Loss When Combined With Denervation

**DOI:** 10.3389/fphys.2020.00445

**Published:** 2020-04-28

**Authors:** Takashi Yamada, Yuki Ashida, Daisuke Tatebayashi, Masami Abe, Koichi Himori

**Affiliations:** Graduate School of Health Sciences, Sapporo Medical University, Sapporo, Japan

**Keywords:** cancer cachexia, denervation, muscle atrophy, catabolism, anabolism

## Abstract

Patients with cancer cachexia (CCX) suffer from muscle wasting, which is often but not always accompanied by selective loss of myosin. Here we examined the effects of CCX on muscle mass and myosin heavy chain (MyHC) expression in denervated (DEN) muscles, especially focusing on the protein synthesis and degradation pathways. Male CD2F1 mice were randomly divided into control (CNT) and CCX groups and their left sciatic nerve was transected. CCX was induced by an intraperitoneal injection of colon 26 cells. After 14 days, the serum concentration of IL-6 and corticosteroid was higher in CCX mice than in CNT mice. The combination of CCX with DEN (CCX + DEN) resulted in a marked reduction of the gastrocnemius muscle weight (−69%) that was significantly lower than DEN (−53%) or CCX (−36%) alone. CCX had no effect on MyHC content, but it elicited a preferential MyHC loss when combined with DEN. The expression levels of autophagy markers cathepsin D and LC3BII/I ratio were markedly higher in the CCX + DEN group than in the CNT + DEN and the CCX groups. Paradoxically, there was an increase in protein synthesis rate and phosphorylation levels of p70S6K and rpS6, markers of mTORC1 signaling, in the CNT + DEN group, and these molecular alterations were inhibited in the CCX + DEN group. Our data indicate that CCX aggravates muscle atrophy in DEN muscles by inducing seletive loss of myosin, which involves inactivity dependent mechanisms that is likely to be a consequence of increased autophagy-mediated protein breakdown coupled with impaired protein synthesis.

## Introduction

Cancer cachexia (CCX) is a multifactorial syndrome characterized by decreased skeletal muscle and adipose tissue mass (Tisdale, 2002). CCX is particularly profound in patients with gastrointestinal, lung, and pancreatic cancers, with about one-thirds of patients losing more than 5% of their baseline body weight (Dewys et al., 1980). This affects quality of life of the patients due to muscle weakness and fatigue and also has been associated with increased susceptibility to chemotherapy toxicity (Andreyev et al., 1998). Unlike starvation, in which adipose tissue is lost while lean body mass is preserved, nutritional supplementation fails to substantially reverse changes in body weight in CCX patients (Brennan, 1977; Evans et al., 1985). Accordingly, muscle wasting and cachexia have long been postulated as key determinants of cancer-related death (Zhou et al., 2010). Therefore, understanding the cellular mechanisms behind the loss of muscle mass during CCX is highly significant from a clinical point of view.

It has been proposed that inflammatory cytokines are fundamental mediators of muscle atrophy in CCX. In cultured myotubes, inflammatory cytokines, including TNF-α, IL-1, and IL-6, directly bind surface receptors on muscle fiber driving muscle atrophy via the regulation of anabolic and catabolic pathways (Acharyya et al., 2004; Li et al., 2005, 2009; Bonetto et al., 2012). On the other hand, a major component of muscle atrophy in response to inflammation *in vivo* has been reported to be the results of the release of glucocorticoids that is likely to be induced by an activation of the hypothalamic-pituitary-adrenal axis (Braun et al., 2011). Strikingly, muscle-specific deletion of the glucocorticoid receptor affords a substantial protection against muscle atrophy associated with tumor growth (Braun et al., 2013).

Myosin is the most abundant protein and comprises about 30% of total muscle proteins. Each myosin molecule is composed of two myosin heavy chains (MyHCs) and four myosin light chains. Several studies have shown a preferential loss of MyHC in the animal models and the patients with CCX (Acharyya et al., 2004; Banduseela et al., 2007; Schmitt et al., 2007; Eley et al., 2008; Ochala and Larsson, 2008), suggesting a critical role of myosin loss in muscle wasting and weakness associated with CCX. Conversely, using the colon 26 (C-26) tumor bearing mice, we and others have demonstrated that all myofibrillar proteins decrease in parallel and that MyHC is not selectively reduced in cachectic muscles (Cosper and Leinwand, 2012; Tatebayashi et al., 2018). Thus, the impact of CCX on the regulation of MyHC expression is still under debate.

Intriguingly, denervation (DEN), and concomintant treatment of dexamethason (DEX), a synthetic glucocorticoid, produces characteristic pathologic feature of severe muscle atrophy, and preferential myosin depletion, although DEX treatment alone elicites muscle atrophy without loss of myosin (Rouleau et al., 1987; Mozaffar et al., 2007; Yamada et al., 2018, 2019). Given that CCX results in increased levels of glucocorticoid (Tanaka et al., 1990), these data imply that not only CCX but also inactivity would be necessary to induce selective loss of myosin, which may explain the conflicting findings regarding the levels of MyHC expression in CCX muscles. Furthermore, although muscle wasting is common end-points of CCX and inactivity, it has not been completely clarified whether these conditions are regulated by different mechanistic drivers. It is crucial therefore to deterimine whether the impacts of combined CCX and inactivity (e.g., DEN) are additive, which could have important clinical ramifications.

Muscle atrophy occurs when the overall rates of protein degradation exceed the rates of protein synthesis. Along with the time-course of muscle atrophy, a rapid loss of MyHC protein and mRNA as well as an activation of ubiquitin-proteasome system (UPS), a major protein degradation pathway, were shown to be induced within 3 days of DEN (Geiger et al., 2003; Sacheck et al., 2007). Despite a progressive decrease in muscle weight, a number of studies have reported a paradoxical increase in total RNA concentration, protein synthesis, and an activation of mTORC1 signaling, a primary translational regulator of muscle protein synthesis, following DEN (Mozaffar et al., 2007; Argadine et al., 2009; Quy et al., 2013). In contrast, DEX treatment does not inhibit MyHC mRNA induction (Mozaffar et al., 2007), but suppress the mTORC1 signaling (Schakman et al., 2013). Moreover, activation of autophagy pathway has been shown to be induced in DEX-treated mouse skeletal muscle (Penna et al., 2013). Hence, a better understanding of the anabolic and catabolic metabolism may provide mechanistic insights regarding the preferential myosin depletion seen in CCX condition.

C-26 tumor bearing mice are a commonly used animal model for CCX (Murphy et al., 2012). Intraperitoneal inoculation of C-26 tumor cells has been used as an animal model for peritoneal metastasis (Matsuyama et al., 2015), whereas C-26 tumor cells are generally inoculated subcutaneously into the flank of mice. Peritoneal metastasis develops in 8.5–25% of patients with colorectal cancer and is associated with severe muscle wasting (Jayne et al., 2002; Hilal et al., 2017). It has been demonstrated that tumor bearing mice inoculated into their intraperitoneal cavity shows a more severe CCX phenotype than those inoculated subcutaneously (Matsuyama et al., 2015). Thus, in the present study, we used the intraperitoneal C-26 tumor bearing mice and tested the following principle hypotheses: the impacts of combined CCX and DEN on muscle mass are additive; a preferential loss of myosin was induced by CCX in combination with DEN, but not CCX alone, and was due to a consequence of accererated protein breakdown concomitant with impaired protein synthesis.

## Materials and Methods

### Ethical Approval

All experimental procedures were approved by the Committee on Animal Experiments of Sapporo Medical University (No. 18-110). Animal care was in accordance with institutional guidelines. A total of 13 mice (Sankyo Labo Service, Sapporo, Japan) were used in these experiments. At the end of the experiment, mice were killed by rapid cervical dislocation under anesthesia with 2% inhaled isoflurane to reach a stable anesthetic plane with consistent breathing rate and no response to toe pinch and muscles were subsequently isolated.

### Experimental Design

Male CD2F1 mice (8 week old, *n* = 13) were supplied by Sankyo Labo Service (Sapporo, Japan) and were randomly assigned into control (CNT) (*n* = 7) and CCX (*n* = 6) groups. Mice were given food and water ad libitum and housed in an environmentally controlled room (24 ± 2°C) with a 12-h light-dark cycle. C-26 cells were provided by the RIKEN BRC through the National Bio-Resource Project of the MEXT, Japan. The C-26 cells were cultured *in vitro* with RPMI-1640 supplemented with 10% (vol/vol) fetal bovine serum and 1% penicillin/streptomycin, and incubated at 37°C with 5% CO_2_. CCX was induced by intraperitoneal injection of 5 × 10^6^ C-26 cells diluted in 0.1 ml phosphate buffered saline and developed for 14 days, as described previously (Matsuyama et al., 2015). At the time of inoculation of C-26 cells, DEN was induced on the left hindlimb (CNT + DEN and CCX + DEN) by removing a 10-mm segment of the sciatic nerve under 2% isoflurane anesthesia. Muscles from the right leg were used as innervated controls. After 14 days, blood samples were collected from the heart under isoflurane anesthesia and centrifuged at 3,000 rpm for 15 min, then serum was separated out and stored at −80°C for later analysis. Mice were killed by cervical dislocation under isoflurane anesthesia and the body weight, including intraperitoneal tumor, was measured. Then, the heart and the plantar flexor muscles were excised from each animal. Of note, it has been demonstrated that intraperitoneal inoculation of C-26 cells develops more rapid and severe CCX compared to subcutaneous inoculation of those cells and that the body weight of intraperitoneal tumor mice starts to decrease significantly around 1 week after inoculation (Matsuyama et al., 2015). Thus, these data suggest the concurrent presense of CCX and DEN at least 1 week before sacrifice in the CCX + DEN group in the present study.

### ELISA

Corticosterone and interleukin (IL)-6 levels were measured in blood serum using corticosterone (No. 501320, Cayman Chemical) and IL-6 (KMC0061, ThermoFisher) ELISA kits according to manufacturer's instructions, respectively. A microplate reader was used to detect optical density of the colorimetric signal. The concentration of corticosterone and IL-6 was calculated by using recombinant mouse corticosterone and IL-6 as a standard, respectively.

### Quantitative Real-Time PCR

Real-time PCR was used to quantify the mRNA levels for regulated in development and DNA damage responses (REDD) 1, forkhead box protein O3 (FoxO3), muscle-specific E3 ubiquitin ligases atrogin-1 and muscle ring finger 1 (MuRF-1) in frozen gastrocnemius (Gas) muscle tissue. Briefly, total RNA was extracted with TORIZOL reagent (Invitrogen, Carlsbad, CA), and the purity and yield of the total RNA extracted was determined by absorbance of aliquots at 260 and 280 nm (Thermo Scientific Nanodrop Light). Total RNA was reverse-transcribed to cDNA using Prime Script RT Reagent Kit (Takara, Japan). Synthesized cDNA was then amplified on the Applied Biosystems 7500 with Premix Ex Taq™ kit (Takara, Japan). The following Taqman Probes (Applied Biosciences™, Carlsbad, CA) were used: mouse REDD1 (Ddit4, Mm00512504_g1), mouse FoxO3 (Mm01185722_m1), mouse atrogin-1 (Fbxo32, Mm00499523_m1), mouse MuRF-1 (Trim63, Mm01185221_m1), mouse TATA (Tbp, Mm00446973_m1). All samples were run in duplicate. Relative amounts of target mRNA was determined using the comparative threshold cycle method (ΔΔCT). Expression of target genes was normalized to the corresponding expression level of TATA.

### Measurement of Protein Synthesis

Muscle protein synthesis rate was measured in Gas muscles using a non-radioactive technique as described previously (Goodman et al., 2011). Briefly, puromycin (0.04 μmol/g body weight dissolved in 100 μl of PBS) was intraperitoneally injected into each animal, and then the Gas muscle was excised and quickly frozen in liquid nitrogen at exactly 30 min following the puromycin injection. The amount of puromycin incorporation into nascent peptide chains was determined by immunoblot analysis (see below).

### Immunoblots

Immunoblots were performed using: anti-actin (A2172, Sigma Aldrich), anti-glutamine synthetase (GS) (GTX109121, GeneTex), anti-cathepsin D (ab75852, Abcam), anti-LC3B (ab63817, Abcam), anti-puromycin (MABE343, Merck Millpore), anti-total 70 kDa ribosomal S6 kinase (p70S6K) (9202, Cell Signaling), anti-p-p70S6K (Thr389) (9205, Cell Signaling), anti-total ribosomal protein S6 (rpS6) (2217, Cell Signaling), and anti-p-rpS6 (Ser240/244) (2215, Cell Signaling).

To extract whole muscle proteins, Gas muscle pieces were homogenized in ice-cold homogenizing buffer (30 μl/mg wet wt) consisting of (mM): Tris maleate, 10; NaF, 35; NaVO_4_, 1; 1% Triton X 100 (vol/vol), and 1 tablet of protease inhibitor cocktail (Roche) per 50 ml. The protein content was determined using Bradford assay (Bradford, 1976). Aliquots of the whole muscle homogenates (15 μg) were diluted with SDS-sample buffer (mM): Tris/HCl, 62.5; 2% SDS (wt/vol); 10% glycerol (vol/vol); 5% 2-mercaptoethanol (vol/vol); 0.02% bromophenol blue (wt/vol). Proteins were separated on 4–15% Criterion TGX Stain Free gels (BioRad). Gels were imaged (BioRad Stain Free imager) and the ratio of MyHC to the total muscle proteins was measured by using Image Lab Software (BioRad). Then proteins were transferred onto polyvinylidine fluoride membranes. Membranes were blocked in 3% (wt/vol) non-fat milk, Tris-buffered saline containing 0.05% (vol/vol) Tween 20, followed by incubation with primary antibody overnight at 4°C. Membranes were then washed and incubated for 1 h at room temperature (~24°C) with secondary antibody (1:10,000, donkey-anti-rabbit or donkey-anti-mouse, BioRad). Images of membrane were collected following exposure to chemiluminescence substrate (Millipore) using a charge-coupled device camera attached to ChemiDOC MP (BioRad), and Image Lab Software was used for detection as well as densitometry.

### Statistics

Data are presented as mean ± SEM. Student's *t*-test was used to compare the body and heart weight and the concentration of corticosteroid and IL-6 between CNT and CCX mice. A two-way ANOVA, followed by the Tukey test for multiple comparisons, was used to determine statistically significant differences in muscle weight and the expression levels of mRNA and protein between the groups. A *P* < 0.05 was regarded as statistically significant. Statistical testing was performed with SigmaPlot (version 13, Systat Software, Inc.).

## Results

### Cancer Cachexia Exacerbates Atrophy in Denervated Mouse Skeletal Muscles

**Figure 1:**
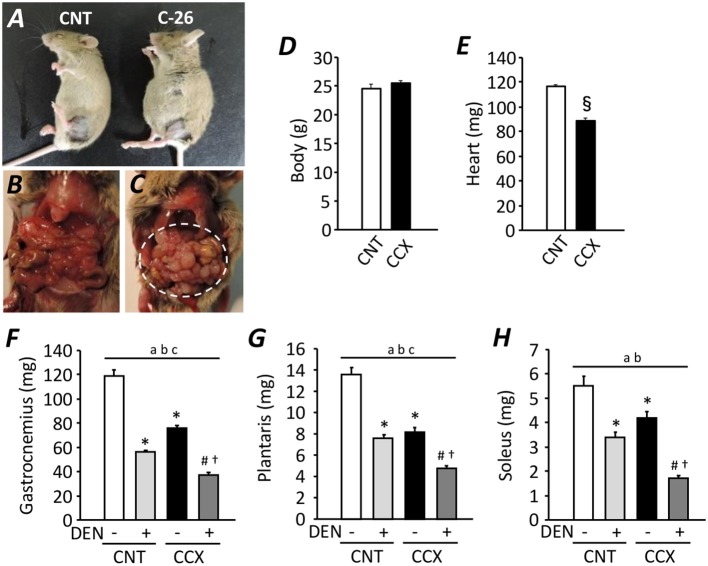
Cancer cachexia exacerbates atrophy in denervated mouse skeletal muscles. Photographs of anesthetized control (CNT) and cancer cachexia (CCX) mice **(A)**. Images after mouse laparotomy in CNT **(B)** and CCX mice **(C)**. Note that CCX mice showed extensive intraperitoneal tumor growth (dotted circle). The body **(D)** and heart **(E)** weight in each group. Bars show the mean and SEM results from 6 to 7 mice per group. ^§^*P* < 0.05 vs. CNT. The muscle wet weight of fast-twitch gastrocnemius **(F)** and plantaris muscles **(G)** and slow-twitch soleus muscles **(H)** from CNT and CCX mice, with or without denervation (DEN). Bars show the mean and SEM results from 6 to 7 muscles per group. Statistical significance was set at *P* < 0.05: main effect of ^a^CCX and ^b^DEN; ^c^interaction of CCX and DEN; difference vs. *CNT without DEN, ^#^CNT with DEN, and ^†^CCX without DEN.

Compared with CNT mice, the serum concentration of corticosteroid and IL-6 was increased in CCX mice [159 ± 4 vs. 236 ± 11 ng/ml (*n* = 5–7), *P* < 0.05; 1.35 ± 0.05 vs. 1.78 ± 0.14 pg/ml (*n* = 5–6), *P* < 0.05, respectively)]. Previous study has shown an increased serum IL-6 concentration as one of the cachexic phenotype, indicating the successful induction of CCX in C-26 tumor bearing mice (Matsuyama et al., 2015). Despite a distended abdomen, a sign of ascites, and extensive intraperitoneal tumor growth in CCX mice (Figures 1A–C), there was no difference in the body weight between the groups (Figure 1D), suggesting reduced tumor-free body mass in CCX mice. The heart weight of C-26 mice was significantly lower (−24%) than that of CNT mice (*P* < 0.05) (Figure 1E). Moreover, the weight for the fast-twitch Gas and plantaris muscles and slow-twitch soleus muscle was decreased by 36, 39, and 24% in the CCX group compared to the CNT group, respectively (*P* < 0.05) (Figures 1F–H). Similar results were obtained in DEN muscles; the weight for the Gas, plantaris, and soleus muscles was reduced by 46, 44, and 39% in the CNT + DEN group compared to the CNT group, respectively (*P* < 0.05). The weight for the Gas, plantaris, and soleus muscle in the CCX + DEN group was decreased by 69, 64, and 69% compared to the CNT group, respectively (*P* < 0.05), and was significantly lower than that of the CNT + DEN and the CCX groups (*P* < 0.05), indicating that the impacts of combined cachexia and inactivity on the regulation of muscle mass are additive.

### Cancer Cachexia Induces Preferential Loss of Myosin in Denervated Mouse Gastrocnemius Muscles

**Figure 2:**
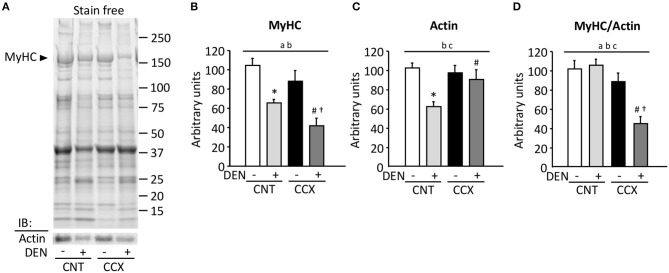
Cancer cachexia induces preferential loss of myosin in denervated mouse gastrocnemius muscles. Representative stain free images of whole muscle proteins and immunoblots for actin in gastrocnemius muscles from control (CNT) and cancer cachexia (CCX) mice, with or without denervation (DEN) **(A)**. Myosin heavy chain (MyHC) **(B)** and actin **(C)** content was normalized by total muscle proteins. The ratio of MyHC to actin **(D)**. Bars show the mean and SEM results from 5 to 6 muscles per group. Statistical significance was set at *P* < 0.05: main effect of ^a^CCX and ^b^DEN; ^c^interaction of CCX and DEN; difference vs. *CNT without DEN, ^#^CNT with DEN, and ^†^CCX without DEN.

Figure 2A shows a typical expression pattern of total muscle proteins and immunoblots for actin in the Gas muscles from each group. Compared to the CNT group, there were significant reductions in the MyHC content in the CNT + DEN (−37%) and the CCX + DEN (−60%) groups (Figure 2B). The degree of reductions in the MyHC content in the CCX + DEN group was much larger than that of the CNT + DEN group. The actin content in the CNT + DEN group was lower than that of the CNT group (Figure 2C). In contrast, there were no significant differences in MyHC and actin content between the CNT and the CCX group. Notably, the ratio of MyHC to actin was markedly lower in the CCX + DEN group than the CNT +DEN and the CCX groups (Figure 2D).

### Cancer Cachexia Increases the Expression Levels of Ubiquitin Ligases mRNA and Autophagy-Related Proteins in Denervated Mouse Gastrocnemius Muscles

**Figure 3:**
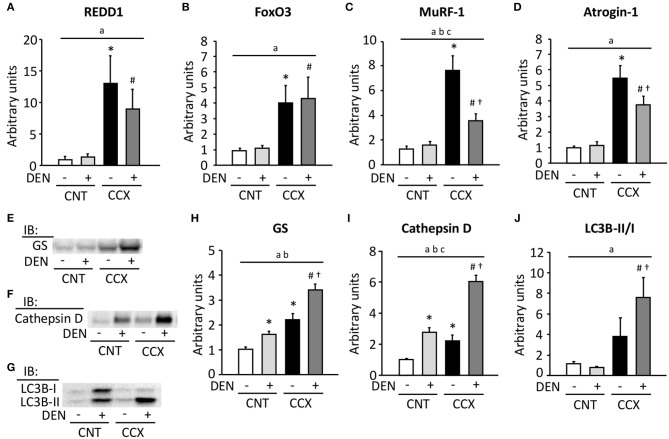
Cancer cachexia increases the expression levels of ubiquitin ligases mRNA and autophagy-related proteins in denervated mouse gastrocnemius muscles. The expression levels of regulated in development and DNA damage responses (REDD) 1 **(A)**, forkhead box O (FoxO) 3 **(B)**, muscle ring finger protein 1 (MuRF-1) **(C)**, and atrogin-1 **(D)** mRNA in gastrocnemius muscles from control (CNT) and cancer cachexia (CCX) mice, with or without denervation (DEN), were normalized to the TATA-binding protein mRNA and expressed as fold change of the mean CNT value, which was set to 1. Representative western blots for glutamine synthetase (GS) **(E)**, cathepsin D **(F)**, and microtube-associated protein 1 light chain 3B (LC3B)-I and -II **(G)**. The expression levels of GS **(H)** and cathepsin D **(I)** were normalized to the total protein content seen in the stain free images. The LC3B-II/LC3B-I ratio **(J)**. Data show mean ± SEM for 5–7 muscles per group. Statistical significance was set at *P* < 0.05: main effect of ^a^CCX and ^b^DEN; ^c^interaction of CCX and DEN; difference vs. *CNT without DEN, ^#^CNT with DEN, and ^†^CCX without DEN.

Expression levels of REDD1, FoxO3, MuRF-1, and atrogin-1 mRNA were markedly increased in Gas muscles from CCX mice compared with CNT mice (Figures 3A–D). Moreover, the expression levels of MuRF-1 and atrogin-1 mRNA in the CCX + DEN group were lower than those of the CCX group. The expression levels of GS and cathepsin D were increased in the CNT + DEN and CCX groups compared with the CNT group and were higher in the CCX + DEN group than those of the CNT + DEN and the CCX groups (Figures 3E,F,H,I). The LC3B-II/I ratio was markedly increasesd in the CCX + DEN group compared to the CNT + DEN and the CCX groups (Figures 3G,J).

### Cancer Cachexia Inhibits the Activation of Protein Synthesis and the mTORC1 Signaling in Denervated Mouse Gastrocnemius Muscles

**Figure 4:**
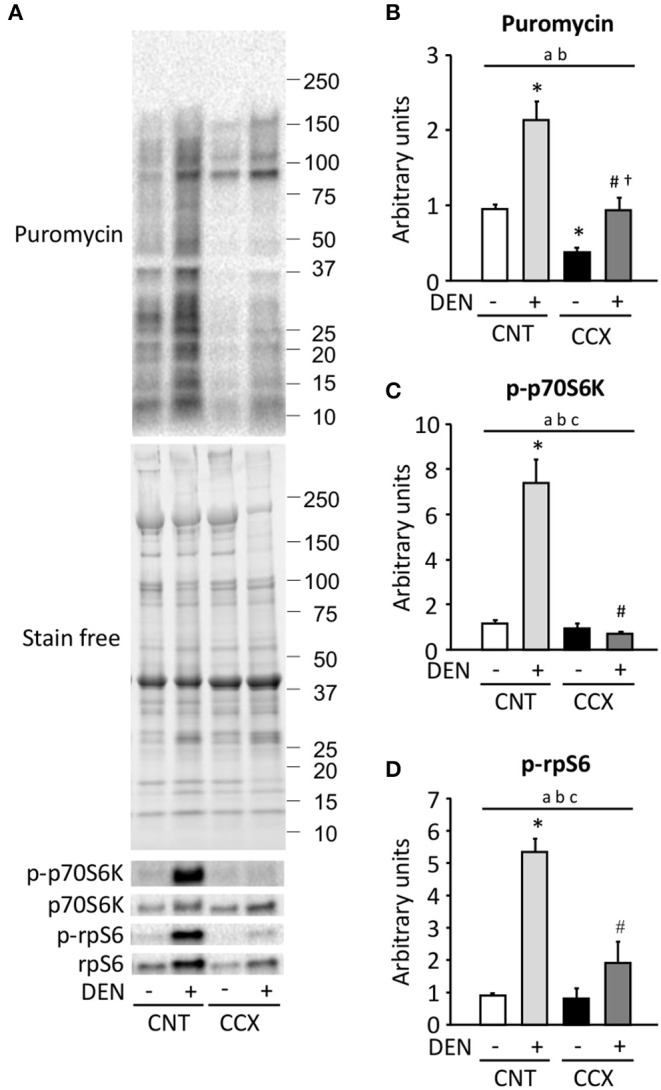
Cancer cachexia inhibits the activation of protein synthesis and the mTORC1 signaling in denervated mouse gastrocnemius muscles. Representative western blots for puromycin, total and phosphorylated p70S6K Thr389 (p-p70S6K), and total and phosphorylated rpS6 Ser240/244 (p-rpS6) in control (CNT) and cancer cachexia (CCX) mice, with or without denervation (DEN) **(A)**. The expression levels of puromycin was normalized to the whole proteins in stain free images and expressed as fold change of the mean CNT value, which was set to 1 **(B)**. The levels of p-p70S6K **(C)** and p-rpS6 **(D)** were normalized to total p70S6K and rpS6 content, respectively. Data show mean ± SEM for 6–7 muscles per group. Statistical significance was set at *P* < 0.05: main effect of ^a^CCX and ^b^DEN; ^c^interaction of CCX and DEN; difference vs. *CNT without DEN, ^#^CNT with DEN, and ^†^CCX without DEN.

Compared to the CNT group, protein synthesis rate, as measured by puromycin incorporation into protein, was increased in the DEN group, while it was reduced in the CCX group (Figures 4A,B). The protein synthesis rate was higher in the CCX + DEN group than in the CCX group. The phosphorylation levels of p70S6K Thr389 and rpS6 Ser240/244 were markedly increased in the DEN group compared to the CNT group (Figures 4A,C,D). Interestingly, both protein synthesis rate and the phosphorylation levels of p70S6K and rpS6 was lower in the CCX + DEN group than in the DEN group.

## Discussion

To our knowledge, this is the first study directly showing that CCX accelerates muscle atrophy in DEN muscles. Moreover, a preferential myosin loss was induced by CCX in combination with inactivity, but not CCX alone, in rodent skeletal muscles. These findings indicate that CCX and inactivity are likely to be regulated by different mechanistic drivers and that CCX-induced selective loss of myosin involves inactivity dependent mechanisms.

The degree of Gas muscle atrophy in the intraperitoneal tumor mice in the present study (−36%) was much greater than that of the subcutaneous tumor mice in our previous study (−11%) (Tatebayashi et al., 2018). These results are consistent with the study by Matsuyama et al. (2015), which showed that the weight loss in Gas muscle is more marked in the intraperitoneal tumor mice than in the subcutaneous tumor mice. Moreover, in line with their findings, myocardium weight loss and increased circulating cytokines were observed in the intraperitoneal tumor mice, suggesting the severe systemic inflammation in those mice. These data suggest that intraperitoneal tumor mice, an animal model for peritoneal metastasis, would be an improved model to study CCX-induced muscle wasting compared to the established subcutaneous tumor mice.

Glucocorticoid appears to be a major determinant of inflammation-induced muscle atrophy and to play an important role in the pathogenesis of CCX (Schakman et al., 2008; Braun et al., 2013). In agreement with the previous studies, we found a marked increase in serum IL-6 (Bonetto et al., 2012) and corticosterone (Tanaka et al., 1990) concentration in CCX mice. FoxO3 has been proposed to regulate autophagy in glucocorticoid-treated and C-26 tumor bearing mice (Penna et al., 2013). Moreover, van der Vos et al. (2012) revealed GS as a FoxO3 target gene that regulate autophagy. It has been demonstrated that a combination of DEN and DEX resulted in additive increases in GS activity (Feng et al., 1990), which is consisitent with our present findings. The mechanism underlying increased GS after DEN appears to be posttranscriptional and is distinct from that of the glucocorticoid-mediated transcriptional regulation of GS induction (Feng et al., 1990). Thus, these data suggest that an activation of glucocorticoid/FoxO3/autophagy network appears to be involved in CCX-induced muscle atrophy.

Paradoxically, protein synthesis, and mTORC1 pathway were activated in DEN muscles, in agreement with what previously reported by others (Argadine et al., 2011; Machida et al., 2012; Quy et al., 2013). It has been suggested that DEN-induced mTORC1 activation is dependent on the proteasome, which is considered to be mediated by amino acids generated from proteasomal degradation (Quy et al., 2013). However, in our study where muscles were denervated for 14 days, activation of mTORC1 signaling was not accompanied by increased mRNA levels of E3 ubiquitin ligases, implying limited intracellular concentration of amino acid at this time point. Thus, although an activation of anabolic signaling raises the possibility that muscle disuse induces compensatory mechanisms to maintain muscle mass and limit atrophy in the absence of innervation (Wang et al., 2005), this may not depend on induction of amino acid by proteasomal degradation. Conversely, CCX suppressed protein synthesis rate, as measured by puromyocin incorporation into protein, which is consistent with our previous study (Tatebayashi et al., 2018). In addition, CCX inhibited increased protein synthesis rate induced by DEN, suggesting that CCX may disrupt compensatory adaptation (i.e., activation of anabolic pathway) in DEN muscles. Thus, although the reduced protein synthesis rate in CCX muscles was restored by DEN treatment, this may not be enough to counteract protein catabolism and hence muscle atrophy was accelerated in DEN muscles from CCX mice.

It is widely accepted that mTORC1 is a major suppressor of autophagy (Kroemer et al., 2010; Mizushima, 2010). Coffer and colleagues (van der Vos et al., 2012) demonstrated that increased levels of GS inhibit mTOR signaling by its lysosomal translocation, which lead to activation of autophagosome formation. Consistent with this, the remarkable increase in GS expression was accompanied by inhibition of DEN-induced hyperphosphorylation of p70S6K and augmentation of LC3BII/I ratio, a marker of autophagosome production, in the CCX + DEN muscles. Additionally, upregulation of cathepsin D, a protease associated with the lysosome autophagy system, implies an increase in autophagosome clearance in the CCX + DEN group. Importantly, previous study has revealed that cathepsin D selectively degrades MyHC in the purified myofibrils from rabbit muscles (Okitani et al., 1981). Thus, these data suggest that CCX aggravates muscle atrophy and preferential myosin loss by activation of autophagy flux in DEN muscles.

Our findings showed that markers of UPS are enhanced in the CCX group in comparison to the CNT + DEN group, while those of autophagy are comparable between these groups. Despite this difference, however, the degree of muscle atrophy was similar between CCX and DEN muscles. This discrepancy may be partly related to the difference in the time-course change in UPS response between DEN and CCX muscles. It has been demonstrated in rat DEN muscles that the two E3 ubiquitin ligases, MuRF1 and atrogin-1, are markedly upregulated at 3 days, when rates of muscle weight loss are the highest, and are returned toward basal levels at 14 days after DEN as the rate of atrophy slowed (Sacheck et al., 2007). These data can explain our findings that neither MuRF1 nor atrogin-1 mRNA were upregulated even though significant wasting was seen in 14 days after DEN. On the other hand, MuRF1 and atrogin-1 mRNA were increased in CCX muscles 14 days after tumor inoculation presumably due to the cachexic phenotype which developed with time in C-26 tumor bearing mice. Suprisingly, despite the upregulation of E3 ubiquitin ligases, CCX alone did not elicite a reduction in myosin content. Although it is well-known that degradation of myofibrillar proteins, such as MyHC, is mainly mediated by the UPS (Sandri, 2008), MuRF1 is regarded as a general ligase affecting many contractile, soluble, and nuclear proteins (Cohen et al., 2009). Therefore, the selective loss of myosin observed in the CCX + DEN muscles is unlikely to be due to the activation of UPS.

Notably, DEN treatment partially suppressed the increased mRNA levels of MuRF-1 and atrogin-1 induced by CCX. Although the reason for these molecular adaptations is unclear, it can be interpreted as an inhibitory effect of DEN on proteasome-mediated muscle protein breakdown in CCX muscles. However, muscle mass was markedly reduced in the CCX + DEN group compared with the CCX group, suggesting that the activation of UPS is not the predominant cause and other pathways are likely to be responsible for the striking decrease in muscle mass in such a situation. In this regard, Wang et al. (2005) have demonstrated that Runx1 (AML1), a DNA-binding protein, is strongly induced in DEN muscles and is required to sustain muscle mass by preventing DEN muscles from autophagy. Interestingly, they suggested that CCX may cause a failure in DEN-induced upregulation of Runx1, resulting in severe muscle wasting accompanied by hallmarks of autophagy. The autophagic-lysosomal pathway has been suggested to be the main proteolytic system in skeletal muscle from non-active cachectic patients with esophageal cancer (Tardif et al., 2013). Thus, these findings further highlight an importance of autophagy pathway in DEN muscles from CCX mice. However, our conclusions regarding mechanism are based on correlations and further studies with pharmacological or genetic inhibition of autophagy at the time of C-26 inoculation and/or DEN would more directly clarify the molecular mechanisms of muscle wasting in the DEN muscles from CCX mice.

Interestingly, DEX treatment has been shown to result in a preferential loss of MyHC in denervated rat skeletal muscle (Rouleau et al., 1987; Mozaffar et al., 2007). A similar picture emerged in our study where selective loss of MyHC was induced by CCX when combined with DEN. Previous study has shown that DEN treatment reduces the expression levels of MyHC and actin mRNA in rat skeletal muscle (Mozaffar et al., 2007). In contrast, our data demonstrated that CCX upregulates REDD1 mRNA and suppresses protein synthesis and mTORC1 pathway in DEN muscles. REDD1 is known to be induced by glucocorticoid and to inhibit anabolic signaling (Britto et al., 2014). Therefore, DEN-induced transcriptional defects coupled with glucocorticoid-induced translational inhibition may be attributed to the preferential myosin depletion in DEN muscles from CCX mice.

## Conclusions

The present results clearly show that the impacts of combined CCX and DEN (i.e., inactivity) on the regulation of muscle mass are additive. Moreover, a preferential myosin loss is induced by CCX when coupled with DEN, but not by CCX alone. Our findings suggest that CCX may disrupt inactivity-induced compensatory mechanisms that preserve muscle mass and limit fiber atrophy, resulting in severe muscle wasting, and selective myosin loss due to increased autophagy-mediated protein breakdown concomitant with impaired protein synthesis.

## Data Availability Statement

All datasets generated for this study are included in the article/supplementary material.

## Ethics Statement

The animal study was reviewed and approved by the Committee on Animal Experiments of Sapporo Medical University (No. 18-110).

## Author Contributions

TY contributed to the conception and design of the study. TY, YA, DT, MA, and KH participated in the analysis and interpretation of the data. TY, YA, and DT were responsible for data collection. TY was involved in writing the manuscript and all authors approved the final version. All authors agreed to be accountable for all aspects of the work in ensuring that questions related to the accuracy or integrity of any part of the work are appropriately investigated and resolved. All persons designated as authors qualify for authorship, and all those who qualify for authorship are listed. All experiments were performed at the Muscle Physiology Laboratory in the Graduate School of Health Sciences, Sapporo Medical University, Sapporo, Japan.

## Conflict of Interest

The authors declare that the research was conducted in the absence of any commercial or financial relationships that could be construed as a potential conflict of interest. The handling editor declared a past co-authorship with one of the authors TY.
